# Safety and outcome of children, adolescents and young adults participating in phase I/II clinical oncology trials: a 9-year center experience

**DOI:** 10.3389/fped.2024.1423484

**Published:** 2024-09-04

**Authors:** Anna Pujol Manresa, Susana Buendía López, Maitane Andión, Blanca Herrero, Álvaro Lassaletta, Manuel Ramirez, David Ruano, Carmen Hernández-Marqués, Amalia Varo, Teresa de Rojas, Marta Cortés Hernández, Jaime Verdú-Amorós, Silvia Martín Prado, Andrea Artigas, Esther Redondo, Julia Ruiz Pato, Pilar Herreros López, Julián Sevilla, Luis Madero, Lucas Moreno, Francisco Bautista Sirvent, Alba Rubio-San-Simón

**Affiliations:** ^1^ Pediatric Hematology-Oncology Department, Hospital Infantil Universitario Niño Jesús, Madrid, Spain; ^2^ Division of Pediatric Hematology and Oncology, Hospital Universitari Vall D'Hebron, Barcelona, Spain; ^3^ La Princesa Institute of Health, Madrid, Spain; ^4^ Pediatric Hematology-Oncology Department, Pediatric Cancer Center Barcelona, Barcelona, Spain; ^5^ ACCELERATE, Brussels, Belgium; ^6^ Pediatric Hematology-Oncology Department, Hospital Regional Universitario de Málaga, Málaga, Spain; ^7^ Pediatric Hematology-Oncology Department, Hospital Clínico Universitario, Valencia, Spain; ^8^ Biomedical Research Institute, INCLIVA, Valencia, Spain; ^9^ Centro de Investigación Biomédica en Red de Cáncer (CIBERONC), Madrid, Spain; ^10^ Pharmacy Department, Hospital Infantil Universitario Niño Jesús, Madrid, Spain; ^11^ Trial and Data Centrum, Princess Máxima Center for Pediatric Oncology, Utrecht, Netherlands

**Keywords:** pediatric hematology and oncology, clinical trials, drug development, clinical research, access to innovation

## Abstract

**Introduction:**

Enrolling children with cancer in early phase trials is crucial to access innovative treatments, contributing to advancing pediatric oncology research and providing tailored therapeutic options. Our objective is to analyze the impact of these trials on patient outcomes and safety, and to examine the evolution and feasibility of trials in pediatric cancer over the past decade.

**Methods:**

All patients recruited in pediatric anticancer phase I/II clinical trials from January 2014 to December 2022 were included. Clinical records and trial protocols were analyzed.

**Results:**

A total of 215 patients (median age 11.2 years, range 1–29.5) were included in 52 trials (258 inclusions). Patients with extracranial solid tumors (67%), central nervous system (CNS) tumors (24%), and leukemia (9%) were included. The most common investigational drugs were small molecules (28.3%) and antibodies (20.5%). Serious adverse events were experienced by 41% of patients, 4.4% discontinued treatment because of toxicity and two had toxic deaths. Median event-free survival was 3.7 months (95%CI: 2.8–4.5), longer in phase II trials than in phase I (2 vs. 6.3 months; *p* ≤ 0.001). Median overall survival was 12 months (95%CI: 9–15), higher in target-specific vs. non-target-specific trials (14 vs. 6 months; *p* ≤ 0.001).

**Discussion:**

A significant and increasing number of patients have been included in early clinical trials, suggesting that both oncologists and families consider it valuable to be referred to specialized Units to access new therapies. Moreover, our data suggests that participation in early clinical trials, although not without potential toxicities, might have a positive impact on individual outcomes.

## Introduction

The outcomes of oncologic pediatric patients have significantly improved over the last decades, partly due to research and development of new anticancer drugs, achieving cure rates above 80% in high-income nations (1, 2). However, pediatric cancer remains the main cause of death among children between the first year of life and adolescence in Western countries and little improvements have been made regarding malignancies with the poorest prognosis, such as advanced or metastatic diseases or certain types of brain tumors, among others (3). Moreover, long-term toxicities of standard anticancer treatments remain a challenge, given that 60%–90% of cancer survivors suffer from drug-related toxicities (4, 5). Research should focus on the development of new anticancer drugs, including treatments based on molecular abnormalities, immunotherapy or combinations (6).

The European Society for Pediatric Oncology (SIOPE), in a joint initiative with patient advocacy groups, delineated in 2021 a strategic plan to develop more effective and less toxic novel therapies for children and adolescents with cancer in Europe, with a view to improve patient outcomes by 2026 (7). For this purpose, recruitment of children in clinical trials needs to be enhanced in order to properly evaluate and test these new drugs, providing robust quality data to subsequently approve their use in the pediatric population.

In the last few years, access to innovative agents for children and adolescents with cancer has notably improved in Spain (8). This outcome stems from modifications in European and Spanish legislations (9, 10), the incorporation of Spanish centers into larger international research partnerships such as the Innovative Therapies for Children with Cancer (ITCC) (11), and the set-up of pediatric-focused initiatives aimed at facilitating the implementation of collaborative clinical trials (12).

The Pediatric Trial Unit at Hospital Niño Jesús (HNJ) (Madrid, Spain) was created in 2013. The main objective was to provide access to new treatments for patients with rare and serious diseases, initially focusing on pediatric oncology trials. The Trial Unit supports investigators from set-up to implementation and conduct of clinical trials. In this manuscript we present an overview of the characteristics, toxicities and outcomes of all children and adolescents with cancer included in phase I/II clinical trials in our center.

## Materials and methods

The analysis comprised all patients included in a phase I or II clinical trial involving anticancer agents from January 2014 to December 2022 at HNJ. The study date cut-off was 1st January 2023. Trials primarily evaluating high-dose chemotherapy and supportive care interventions were not included. Patients who signed informed consent were included even if they were subsequently considered screening failures. Patients were included at the time of their first or subsequent participation in an early phase clinical trial (ECT). Patient data were reported through revision of medical records. Institutional approval for retrospective chart review was obtained.

Trial and investigational medical products (IMPs) information was extracted from trial protocols. Dose-limiting toxicities (DLTs) and response to study treatment definitions were assessed according to each protocol. Adverse events grading and assessment of casualty were made by the investigators. Toxicities were included in the analysis even if the investigator did not consider it related to the IMP and graded according to CTCAE. Distance from the patient’s town to HNJ was estimated using a standard online route planner (13).

Clinical trials were classified either as phase I studies, restricted to those with only a dose-finding/dose confirmation component, or as phase II studies, including trials with a transition phase (phase I/II) and “strict” phase II trials. Multi-arm trials were considered as one single trial for this analysis.

Tumor-specific trials were defined as trials focusing exclusively on histological tumor types; while target-specific trials were defined as trials in which inclusion criteria were determined by the molecular target. Median time on trial and event-free survival (EFS) were calculated from date of consent to death from any cause or progressive disease for patients that underwent study intervention. Overall survival (OS) was calculated from date of consent to death from any cause or date of last follow up. EFS and OS were analyzed using Kaplan-Meier curves, and comparisons were made by log-rank formulas. For the univariate analysis, *p* values less than 0.05 were considered statistically significant.

## Results

### Trials’ description

During the study period, 62 anticancer ECT were open and 52 (84%) recruited patients. Out of the 52 trials, 28 were phase I trials and 24 phase II trials. The list of clinical trials is presented in the Supplementary Material.

**Figure 1:**
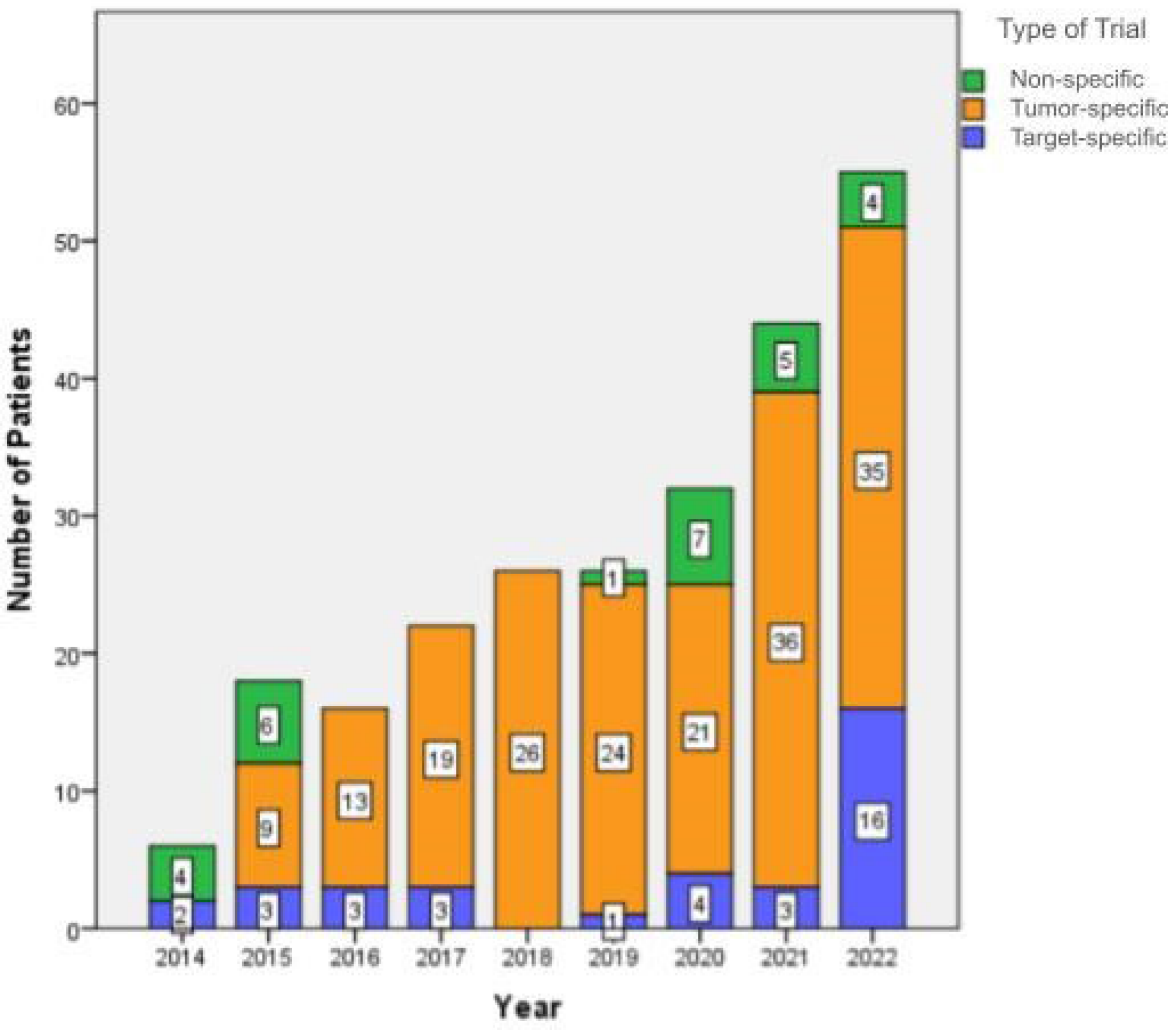
Recruitment of patients in clinical trials from 2014 to 2022. The recruitment is shown per year and by trial type (non-specific trial, tumor-specific trial, and target-specific trial).

From the 52 ECT recruiting trials there were 8 target-specific trials (15%), 39 tumor-specific (75%), and 5 were non-specific trials (10%), with a significant increase in the recruitment of patients in target-specific trials during the last year (Figure 1).

The most frequent type of IMPs were small molecules (35%), followed by cytotoxic agents (17%). Detailed information regarding the type of IMPs is summarized in Table 1.

**Table 1:** Characteristics of trials and the included population.

	Total of trials *n* (%)	Phase I trials *n* (%)	Phase II trials *n* (%)
Trials’ characteristics	*n* = 52 (100)	*n* = 28 (54)	*n* = 24 (46)
Sponsor			
Academic	14 (27)	7 (25)	7 (29.2)
Industry	38 (73)	21 (75)	17 (70.8)
Design			
Non-specific	5 (10)	4 (14)	1 (4.1)
Tumor-specific	39 (75)	18 (65)	21 (87.5)
Target-specific	8 (15)	6 (21)	2 (8.4)
IMP			
Single small molecule	16 (31)	9 (32)	7 (29.2)
>1 small molecules	2 (4)	1 (4)	1 (4.1)
Single monoclonal antibody	6 (11)	3 (10)	3 (12.5)
>1 monoclonal antibodies	2 (4)	1 (4)	1 (4.1)
Cytotoxic agents	9 (17)	2 (7)	7 (29.2)
Cytotoxic drug + small molecules	9 (17)	6 (21)	3 (12.5)
Oncolytic virus	2 (4)	2 (7)	0 (0)
Cytotoxic drug + antibodies	4 (8)	2 (7)	2 (8.4)
Cytotoxic drug + oncolytic virus	1 (2)	1 (4)	0 (0)
Cell therapy	1 (2)	1 (4)	0 (0)
Population’ characteristics	*n* = 258 (100)	*n* = 148 (57)	*n* = 110 (43)
Sex			
Male	151 (58)	86 (58)	65 (59)
Female	107 (42)	62 (42)	45 (41)
Age at inclusion (years)			
<2	4 (1.6)	2 (1.3)	2 (2)
2–5	40 (15.5)	12 (8)	28 (25.5)
6–11	100 (39)	61 (41.2)	39 (35.5)
12–17	86 (33.4)	55 (37)	31 (28)
18–24	14 (5)	6 (4)	8 (7)
>24	1 (0.5)	1 (0.5)	0 (0)
Screening failures	13 (5)	11 (7.5)	2 (2)
Referring hospital			
Patients from Hospital Niño Jesus	81 (31.5)	42 (28.5)	39 (35.5)
Other hospital in Madrid	54 (21)	28 (19)	26 (23.5)
Other hospital in Spain	122 (47)	77 (52)	45 (41)
International referral	1 (0.5)	1 (0.5)	0 (0)
Disease status at inclusion			
Diagnosis	11 (4.5)	4 (2.7)	7 (6.5)
Relapsed disease	169 (65.5)	99 (66.9)	70 (63.5)
Refractory disease	60 (23)	41 (27.7)	19 (17)
Continuation/consolidation therapy	18 (7)	4 (2.7)	14 (13)
Tumor type			
Brain tumors:	61 (23.5)	28 (18.8)	33 (30)
Medulloblastoma or supratentorial PNET	17 (7)	8 (5.5)	9 (8)
High grade glioma	15 (5.8)	9 (6)	6 (5)
DIPG	7 (2.7)	6 (4)	1 (1)
Ependymoma	6 (2)	2 (1.3)	4 (4)
Other central nervous system tumors	16 (6)	3 (2)	13 (12)
Extracranial solid tumors:	161 (62.5)	97 (65.4)	64 (58)
Neuroblastoma	43 (17)	15 (10)	28 (25.5)
Ewing sarcoma	38 (15)	19 (13)	19 (17)
Osteosarcoma	37 (14)	29 (19)	8 (7)
Rhabdomyosarcoma	14 (5)	12 (8)	2 (2)
Wilms tumor	7 (2.7)	7 (5)	0 (0)
Non-rhabdomyosarcoma soft tissue sarcoma	5 (1,9)	3 (2)	2 (2)
Carcinoma	5 (1,9)	4 (3)	1 (1)
Melanoma	2 (1)	2 (1.3)	0 (0)
Germ-cell tumors	2 (1)	1 (0.6)	1 (1)
Other solid tumors	8 (3)	5 (3.5)	3 (2.5)
Hematological tumors:	36 (14)	23 (15.8)	13 (12)
Acute lymphoblastic leukemia	13 (5)	11 (7.5)	2 (2)
Lymphoma	12 (4.6)	3 (2)	9 (8)
Acute myeloid leukemia	7 (2.7)	7 (5)	0 (0)
Chronic myeloid leukemia	4 (1.6)	2 (1.3)	2 (2)
Prior treatment received			
No previous treatment	31 (12)	10 (6.7)	21 (19)
1 prior line	85 (33)	30 (20.3)	55 (50)
2 prior lines	62 (24)	42 (28.4)	20 (18)
3 prior lines	41 (16)	34 (23)	7 (6.5)
4 prior lines	22 (8.5)	18 (12.2)	4 (4)
≥5 prior lines	17 (6.5)	14 (9.4)	3 (2.5)
Prior inclusions in clinical trials			
1st inclusion	207 (80)	110 (74.3)	97 (88)
2nd inclusion	40 (15.5)	29 (19.6)	11 (10)
3rd inclusion	9 (3.5)	7 (4.7)	2 (2)
4th inclusion	2 (1)	2 (1.4)	0 (0)
Design of the trial			
Non-specific	27 (10.5)	25 (16.9)	2 (2)
Tumor-specific	194 (75)	88 (59.5)	106 (96)
Target-specific	37 (14.5)	35 (23.6)	2 (2)
Investigational products received			
Single small molecule	58 (22.5)	37 (25)	21 (19)
>1 small molecule	15 (5.8)	13 (9)	2 (2)
Single monoclonal antibody	43 (17)	20 (13.3)	23 (21)
>1 monoclonal antibodies	10 (3.7)	7 (4.7)	3 (2.5)
Cytotoxic agents	51 (20)	13 (9)	38 (34.5)
Cytotoxic drug + small molecules	43 (17)	30 (20)	13 (12)
Oncolytic virus	12 (4.5)	12 (8)	0 (0)
Cytotoxic drug + antibodies	12 (4.5)	2 (1.5)	10 (9)
Cytotoxic drug + oncolytic virus	9 (3)	9 (6)	0 (0)
Cell therapy	5 (2)	5 (3.5)	0 (0)

PNET, primitive neuroectodermal tumor; DIPG, diffuse intrinsic pontine glioma; IMP, investigational medicinal product.

There was a median of five patients included per trial (range 1–19). Interestingly, over the last two years (from January 2021 to December 2022) the recruitment increased substantially, accounting for 38.4% (*n* = 99) of all the reported inclusions (Figure 1).

### Patient population

The main patient characteristics are shown in Table 1. A total of 215 patients were included. There were 28 patients included in two trials, 6 in three trials and 1 patient in four, resulting in 258 inclusions. Out of the 258 inclusions, 148 (57.3%) and 110 (42.6%) occurred in phase I and II trials respectively. Out of those, there were 11 (7.4%) and two (1.8%) screening failures respectively due to not meeting the eligibility criteria of the trials.

Median age at diagnosis was 8.7 years (range 0.2–19.6) and median age at consent was 11.2 years (range 1–29.5). Most patients were adolescents and young adults between the ages of twelve and twenty (43.7%), and only 4 patients (1.6%) were aged below two years at inclusion.

Disease status at enrollment was relapsed or refractory disease in 229 cases (88.7%) and at initial diagnosis in eleven (4.2%). Most patients had received prior treatment with chemotherapy (88.4%), with a median of 2 regimens before trial entry (range 0–7).

Of the 258 inclusions, the majority were referred for ECT from hospitals in other 28 Spanish cities (47%, *n* = 122) or from other hospitals within the Madrid region (21%, *n* = 54). There was only one patient that was referred from a different country (Portugal). Median travel distance for patients coming from other cities was 400 km (range 100–2,000).

### Outcomes

**Figure 2:**
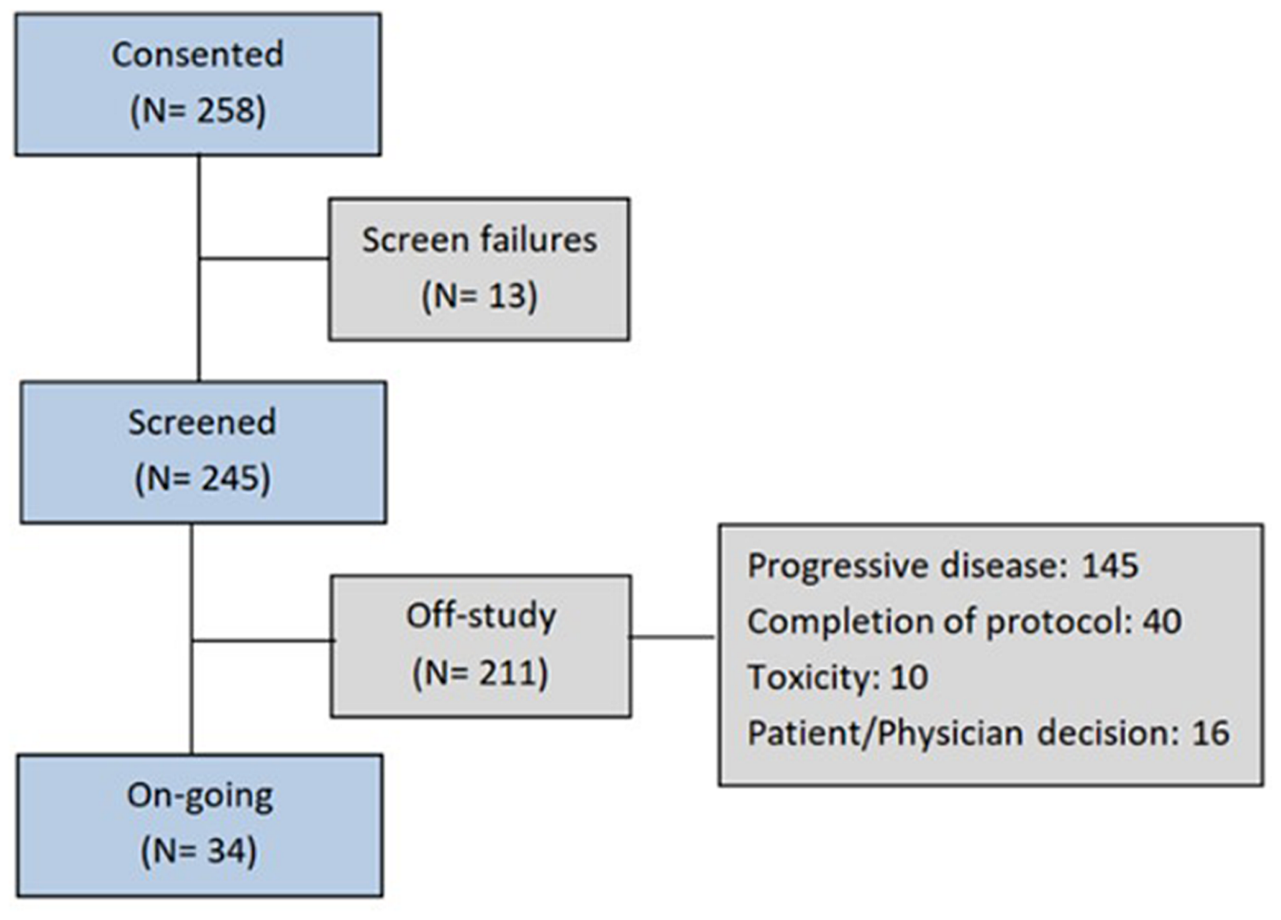
Patient flow through the trial.

The median time on trial was 2.6 months (range 4 days–7.4 years), longer in phase II trials (median of 4 months, range 6 days–5.2 years) than in phase I (median of 1.9 months, range 4 days–7.4 years). Thirty-four patients were under treatment at the time of data cutoff. Of the 211 occasions where patients came off study having received the study drug, the most common reason was disease progression (*n* = 145; 69%). See flow diagram in Figure 2.

The overall response rate (ORR) was 18.2% for phase I trials (12 complete responses and 13 partial responses) and 24.1% for phase II trials (17 complete responses and 9 partial responses). Up to 35% (*n* = 86) of the treated subjects experienced disease stabilization during a median of 5.2 months (range 1–46.6). ORR varied between solid and leukemia trials. In phase I trials, patients with hematologic malignancies had an ORR of 68.7% compared to 11.6% for those with solid tumors. In phase II, patients with hematologic malignancies showed an ORR of 77% and an ORR of 16.8% for patients with solid tumors.

**Figure 3:**
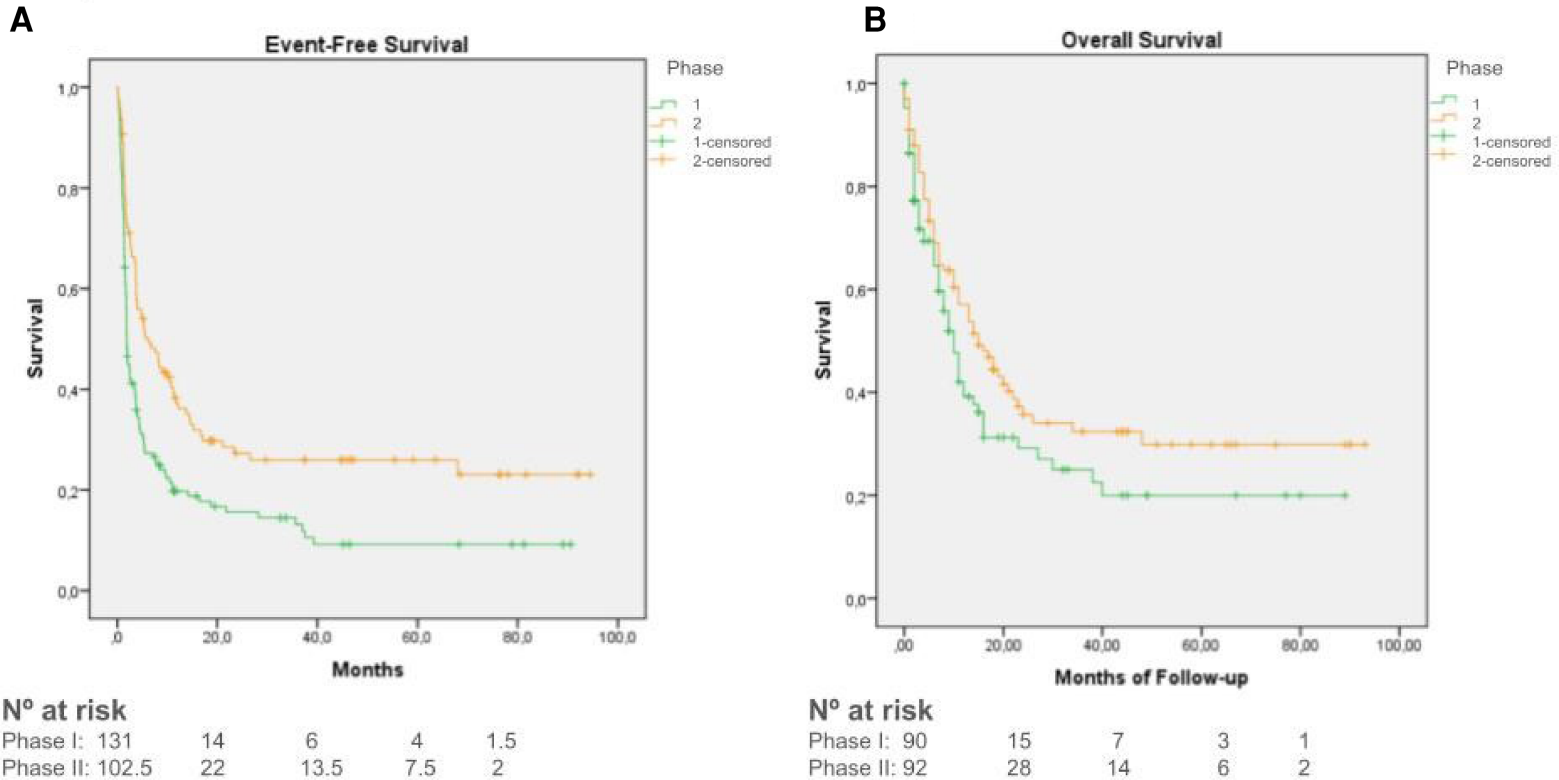
**(A)** Kaplan–Meier curves for event-free survival of patients enrolled in phase I clinical trials (green) and phase II clinical trials (orange). **(B)** Kaplan–Meier curves for Overall Survival of patients enrolled in phase I clinical trials (green) and phase II clinical trials (orange).

**Figure 4:**
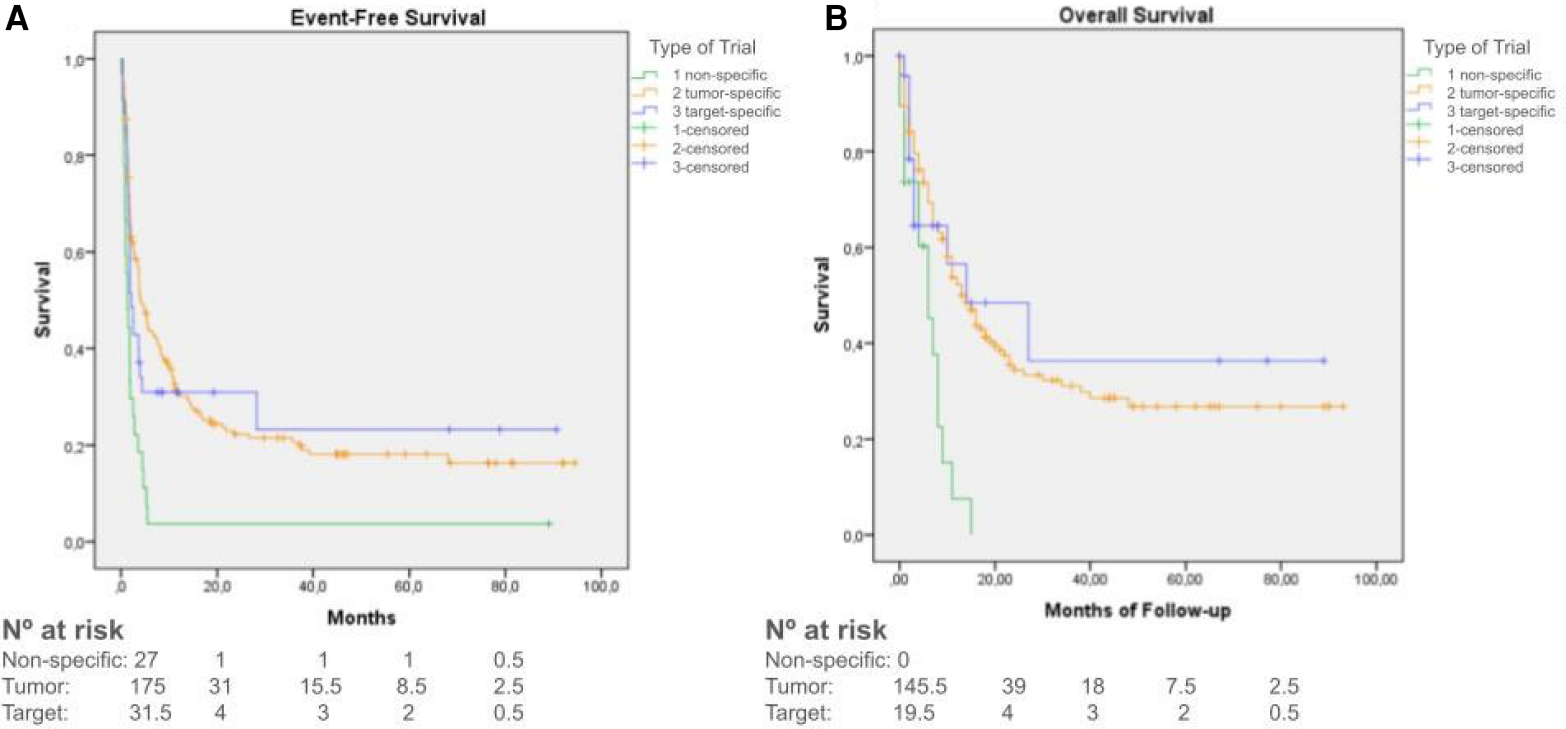
**(A)** Kaplan–Meier curves for event-free survival of patients enrolled in non-specific trials (green), tumor-specific trials (orange) and trials using target-specific therapies (blue). **(B)** Kaplan–Meier curves for Overall Survival of patients enrolled in non-specific trials (green), tumor-specific trials (orange) and trials using target-specific therapies (blue).

Median EFS for all patients was 3.7 months (95%CI: 2.8–4.5 months). Median EFS in phase I trials was 2 months (95%CI: 1.4–2.4), while in phase II EFS was 6.3 months (95%CI: 2.7–9.8) (Figure 3A). Data showed better results of EFS for patients being included in a tumor or target-specific therapeutic trials (median EFS of 4 and 2 months respectively, 95%CI 2.9–5.3 and 1.5–2.7), when compared to non-specific trials (median EFS of 1.4 months, 95%CI 0.8–1.9) (Figure 4A).

**Figure 5:**
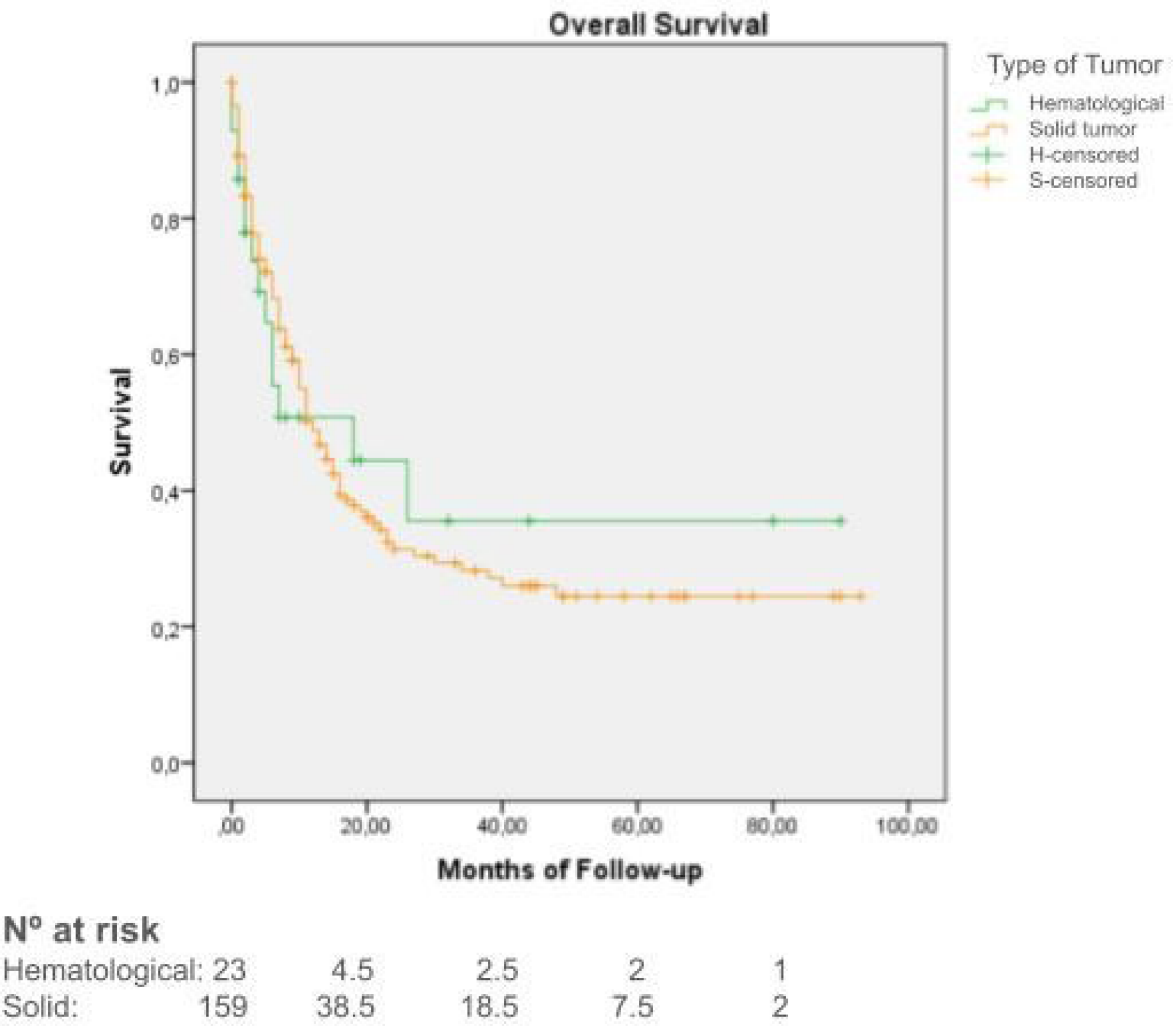
Kaplan–Meier curves overall survival of patients with hematological malignancies (green) vs. solid tumors (orange).

Median OS was 12 months (95%CI: 9–15 months), with longer survival in phase II trials (median OS of 15.6 months, 95%CI 9.3–20.6) compared to phase I trials (median OS of 10.1 months, 95%CI 7.6–12.3) (*p* = 0.073) (Figure 3B). Data also showed better results of OS in target-specific trials (median OS of 14 months, 95%CI 0–34.5) when compared to non-specific trials (median OS 6 months, 95%CI 2.6–9.3) (*p* < 0.001) (Figure 4B). Median OS for patients with hematologic malignancies was 18 months (95%CI 0–41), and 12 months (95%CI 9–15) for those with solid tumors (*p* = 0.01) (Figure 5).

### Safety

Among all 258 inclusions, 162 (62.8%) experienced grade 3 adverse events (AE) and 75 (29.1%) reported grade 4 AEs. The most common grade 3/4 AEs were hematologic, comprising 68% of grade 3% and 85% of grade 4 AEs. Among non-hematologic AEs reported, the most frequent was hypertransaminasemia (23% of grade 3% and 7% of grade 4 AEs), followed by pain, diarrhea and skin rash. Grade 5 events (death) were reported in two patients. There was a higher incidence of grade 3 and 4 AEs in phase II trials compared to phase I (73.6% and 31.8% in phase II vs. 54.7% and 27% in phase I). Among phase I studies there were 12 DLT, representing 8.1% of the inclusions.

As for severe adverse events (SAEs), there were 106 notifications in 41% of the recruited patients; 55 (37.2%) were reported in phase I trials and 51 (46.4%) in phase II trials.

There were 121 registered deaths among treated patients, mainly caused by progressive disease (94.2%; *n* = 114). There were 4.1% (*n* = 5) of unknown/unclassified causes and 1.6% (*n* = 2) caused by drug toxicity.

## Discussion

This is the largest report on a series of children and adolescents with malignancies treated in a single institution in Spain within phase I or II anticancer trials. Our results show that participation in pediatric oncology ECT can have a positive impact on individual outcomes. Our experience confirms that it is possible to promote the development of new antineoplastic drugs for childhood across the country.

HNJ Clinical Trials Unit's success stems from strategic competitive funding, initiated in 2013 with a Ministry of Health Grant, aiding in staff recruitment and infrastructure setup. Joining ITCC in 2014 expanded capabilities and collaborations, leading to ITCC Early Phase accreditation in 2017. The emphasis on securing competitive funding and hospital recognition enabled the unit to excel in pediatric oncology research despite challenges, including COVID-19, demonstrating resilience and robust recruitment. As one of the largest Pediatric Early Phase Clinical Trial Units in Europe, our experience can serve as a valuable reference for oncologists globally who are seeking to establish a new Early Phase Clinical Trial Unit and accurately depicts the landscape of contemporaneous clinical research in pediatric oncology. This may provide useful information to generate a baseline dataset and may also encourage similar studies that would allow for a bigger-scale descriptive analysis at a national and international level.

Collaborative clinical trials are one of the main contributors to the improvement in survival for many childhood cancers (14). As stated in the document *European Standards of Care for Children with Cancer*: “When available, children should be offered the opportunity to participate in relevant clinical trials that aim to improve the optimal treatment for all children” (15). Historically, Spain has not been able to participate in multiple international clinical trials because of lack of resources and infrastructure to adapt to modern regulatory standards (12). However, the number of pediatric cancer ECT, and consequently the number of enrolled patients in Spain, has increased notably over the last decades (8, 16, 17). The total number of trials available increased 4.4-fold over the period 2014–2020 compared to 2007–2013 (8). This is consistent with the reported data in our center, where during the last two years the number of available trials increased, but also the amount of included patients. The participating population encompassed patients with a wide array of pediatric cancer conditions. However, it is worth noting that patients with hematological malignancies constitute only 9.3% (*n* = 24) of the recruited patients, even though acute leukemia is the most common childhood cancer (18). This lack of representation is a concern that has been raised by other research groups internationally and measures to cover this unmet need must be implemented (19).

Setting up ECT requires significant resources and specialized expertise. Consequently, these trials may only be available in a few centers, potentially obliging patients to undertake long journeys to reach the trial site. Our findings emphasize that families and patients are willing to travel (even considerable distances) to access novel therapies, although strategies to limit the burden of participation into clinical trials need to be implemented, in order to improve equity of access regardless of geographical situation (20). Moreover, the high accrual of patients from different centers across Spain suggests that oncologists throughout the territory recognize the importance, and potential benefits of having access to clinical trials. These results go in consonance with the British experience ublished by the Royal Marsden group (19). The development of clinical research networks, tools to support families needing to travel and deeper involvement of patient advocacy organizations would further facilitate patient referrals.

In line with the national-level reporting and the experience of other large European pediatric clinical trial units, the majority of our patients were enrolled in industry-sponsored trials (66%) (8, 19, 21). This is likely due to an increased activity of commercial sponsors in Spain. The pharmaceutical industry has increased its investing in clinical research since 2005 (22). In addition, the 2006 European Pediatric Regulation requires an agreed pediatric investigation plan before marketing authorization is given for adult medicines (10). This regulation has encouraged pharmaceutical companies to embrace drug development for childhood cancer (23). Still, further efforts are needed to enhance our ability to deliver academic trials, both local as well as international trials from ITCC and other networks that need support to open in Spain.

In our center, biomarker-guided approach accounted for 14.3% of the recruitments, which is in line with the experience from other groups and shows how the landscape of ECT in pediatric oncology is evolving (24). Our data showed better results for patients in biomarker driven studies in terms of survival, as it was already suggested in the adult population (25). However, other reports such as the German INFORM registry showed that this benefit was restricted to patients with highly relevant oncogenic drivers (26). Moreover, it is important to note that these molecularly driven trials are addressed to only a small proportion of patients and in Spain, genomic testing in pediatric cancer is still not widely available (27).

Regarding outcomes and toxicities of pediatric patients enrolled in ECT, data is scarce, both at a national and international level. However, our data are consistent with previous reports, and suggests that participation in ECT, although not without potential toxicities, might have a positive impact on individual outcomes, mainly in terms of disease stabilization (19, 21). Our ORR, both in phase I (18.2%) and phase II (24%) trials show a modest increase over time, when compared to the previously reported data in other international centers (ORR of 4%–15% in phase I and 12%–12.9% for phase II trials) (19, 21, 28–32). However, the populations are not entirely comparable. For instance, the experience at Gustave-Roussy Hospital only includes patients with solid and brain tumors, in which the prognosis is poorer than in hematological tumors (21). Additionally, small differences in the number of patients with certain diseases, such as low-grade glioma, where the nature of the tumor often leads to disease stabilization rather than a response, can result in significant changes in the global ORR. Another cause to be considered is the difference in the studied years, as the previously reported data are from at least a decade ago. The greater biological insight in pediatric tumors and the novel trial designs may have had a potential clinical impact (33, 34). Another result worth highlighting is the OS obtained, particularly in phase II trials. It is important to consider that the main driving force for patients with advanced cancer and their families to participate in an experimental phase trial is the expectation of experiencing clinical benefits (35). Nevertheless, most pediatric ECTs have not included measures of clinical benefit such as symptom relief, quality of life and disease stabilization (28, 32). Up to 35% of our subjects experienced disease stabilization, with some exceptional responders remaining on trial beyond three years. Given that most of our patients had advanced disease at the outset of the study, the positive impact of disease stabilization on clinical outcomes should not be overlooked.

Additionally, the majority of subjects had a good safety profile despite extensive pretreatment. The DLT rate of 8.1% is comparable with previously published reports (19, 21, 29, 30). This could support challenging traditional trial designs, switching from a dose-finding to a dose-confirmation approach of ECT in pediatrics (starting upfront with the 100% of the body surface area-adjusted adult dose to children), in order to speed up pediatric drug development (36, 37). The toxic death rate was 1.6%, which is similar to the previously published for pediatric ECTs (19, 21). However, consideration should be given to the percentage of grade 3–4 toxicities that notably affect the patient's quality of life or hinder the administration of the study drug.

In conclusion, this study offers valuable insights into ECT for childhood cancer, emphasizing a notable rise in children and adolescents’ participation over the past decade. It underscores the shared acknowledgment between oncologists and families regarding the importance of referral to specialized units for accessing innovative therapies. We show that ECT can provide reasonable life expectancy for pediatric oncology patients, mostly in target and tumor-specific trials, even though they are not exempt from suffering drug-related adverse events. Specialized clinical trial units have the mission to ensure access to new therapies, and to obtain data on efficacy and safety that will enable the development of new therapeutic strategies to improve outcomes for these patients in the near future. Our experience can serve as a valuable reference for pediatric oncologists globally who are seeking to establish Early Phase Clinical Trial Units.

## Data Availability

The original contributions presented in the study are included in the article/Supplementary Material, further inquiries can be directed to the corresponding author.

## References

[1] Allemani C Matsuda T Di Carlo V Harewood R Matz M Nikšić M Global surveillance of trends in cancer survival 2000–14 (CONCORD-3): analysis of individual records for 37 513 025 patients diagnosed with one of 18 cancers from 322 population-based registries in 71 countries. Lancet. (2018) 391:1023–75. 10.1016/S0140-6736(17)33326-3 29395269 PMC5879496

[2] Smith MA Altekruse SF Adamson PC Reaman GH Seibel NL. Declining childhood and adolescent cancer mortality. Cancer. (2014) 120(16):2497–506. 10.1002/cncr.28748 24853691 PMC4136455

[3] Smith MA Seibel NL Altekruse SF Ries LA Melbert DL O'Leary M Outcomes for children and adolescents with cancer: challenges for the twenty-first century. J ClinOncol. (2010) 28(15):2625–34. 10.1200/JCO.2009.27.0421 PMC2881732 20404250

[4] Bleyer WA. Cancer in older adolescents and young adults: epidemiology, diagnosis, treatment, survival, and importance of clinical trials. Med PediatrOncol. (2002) 38(1):1–10. 10.1002/mpo.1257 11835231

[5] PDQ Pediatric Treatment Editorial Board. Late Effects of Treatment for Childhood Cancer (PDQ®): Health Professional Version. In: PDQ Cancer Information Summaries. US: National Cancer Institute (2023). 26389273

[6] Zhong L Li Y Xiong L Wang W Wu M Yuan T Small molecules in targeted cancer therapy: advances, challenges, and future perspectives. Signal Transduct Target Ther. (2021) 6:201. 10.1038/s41392-021-00572-w 34054126 PMC8165101

[7] SIOPE. SIOP Europe Strategic Plan. Brussels, Belgium: A European Cancer Plan for Children and Adolescents (2021).

[8] Rubio-San-Simón A HladunAlvaro R Juan Ribelles A Castañeda Heredia A Guerra-García P Verdú-Amorós J The paediatric cancer clinical research landscape in Spain: a 13-year multicentre experience of the new agents group of the spanish society of PaediatricHaematology and oncology (SEHOP). Clin Transl Oncol. (2021) 23(12):2489–96. 10.1007/s12094-021-02649-y 34076861

[9] Boletín Oficial del Estado. ES_RD_1090/2015_Ensayos Clínicos. España: Bol Of Del Estado (2015). p. 121923–64.

[10] Unión Europea. Reglamento (CE) NO1901/2006 del Parlamento Europeo y del Consejo de 12 de Diciembre de 2006 Sobre Medicamentos para uso Pediátrico y por el que se Modifican el Reglamento (CEE) no1768/92, la Directiva 2001/20/CE, la Directiva 2001/83/CE y el Reglamento (CE) 2006.

[11] Zwaan CM Kearns P Caron H Verschuur A Riccardi R Boos J The role of the “innovative therapies for children with cancer” (ITCC) European consortium. Cancer Treat Rev. (2010) 36(4):328–34. 10.1016/j.ctrv.2010.02.008 20231057

[12] Bautista F Cañete A Ramírez-Villar GL Fernández JM Fuster JL de Heredia C D ECLIM-SEHOP, a new platform to set up and develop international academic clinical trials for childhood cancer and blood disorders in Spain. Clin Transl Oncol. (2019) 21(12):1763–70. 10.1007/s12094-019-02221-9 31598904

[13] Google. Google maps. Available online at: https://www.google.com/ (Accessed February 15, 2023).

[14] Kowalczyk JR Samardakiewicz M Pritchard-Jones K Ladenstein R Essiaf S Fitzgerald E European Survey on standards of care in paediatric oncology centres. Eur J Cancer. (2016) 61:11–9. 10.1016/j.ejca.2016.03.073 27131152

[15] SIOP. European Standards of Care for Children with Cancer. Brussels, Belgium: SIOP Europe (2009). p. 1–27.

[16] Bautista F Gallego S Cañete A Mora J Díaz de Heredia C Cruz O Early clinical trials in paediatric oncology in Spain: a nationwide perspective. An Pediatría (Barc). (2017) 87(3):155–63. 10.1016/j.anpedi.2016.07.007 28279690

[17] Bautista F Gallego S Cañete A Mora J de Heredia CD Cruz O Landscape of early clinical trials for childhood and adolescence cancer in Spain. ClinTranslOncol. (2016) 18(7):708–13. 10.1007/s12094-015-1421-9 26489424

[18] Pardo Romaguera E Muñoz López A Valero Poveda S Porta Cebolla S CañeteNieto A Barreda Reines MS Cáncer Infantil en España. Estadísticas 1980–2021. Registro Español de Tumores Infantiles (RETI-SEHOP). España: Ministerio de Sanidad, Servicios Sociales e Igualdad (2022).

[19] Morgenstern DA Hargrave D Marshall L V Gatz SA Barone G Crowe T Toxicity and outcome of children and adolescents participating in phase I/II trials of novel anticancer drugs: the royal marsden experience. J PediatrHematolOncol. (2014) 36(3):218–23. 10.1097/MPH.0000000000000003 24322496

[20] European Medicines Agency (EMA). Recommendation Paper on Decentralized Elements in Clinical Trials. Brussels, Belgium: European Commission (2022). p. 1–33.

[21] Bautista F Di Giannatale A Dias-Gastellier N Fahd M Valteau-Couanet D Couanet D Patients in pediatric phase I and early phase II clinical oncology trials at gustave roussy: a 13-year center experience. J PediatrHematolOncol. (2015) 37(2):e102–10. 10.1097/MPH.0000000000000237 25171452

[22] Farmaindustria. La industria farmacéutica ha duplicado su inversión en investigación clínica en españa desde 2005. Nota de Prensa. (2019):1–3.

[23] Toma M Felisi M Bonifazi D Bonifazi F Giannuzzi V Reggiardo G Paediatric medicines in Europe: the paediatric regulation—is it time for reform? Front Med. (2021) 8:1–9. 10.3389/fmed.2021.593281 PMC7884470 33604345

[24] Roper N Stensland KD Hendricks R Galsky MD. The landscape of precision cancer medicine clinical trials in the United States. Cancer TreatRev. (2015) 41(5):385–90. 10.1016/j.ctrv.2015.02.009 25864024

[25] Dietel M Jöhrens K Laffert M V Hummel M Bläker H Pfitzner BM A 2015 update on predictive molecular pathology and its role in targeted cancer therapy: a review focussing on clinical relevance. Cancer Gene Ther. (2015) 22(9):417–30. 10.1038/cgt.2015.39 26358176

[26] Van Tilburg CM Pfaff E Pajtler KW Langenberg KPS Fiesel P Jones BC The pediatric precision oncology inform registry: clinical outcome and benefit for patients with very high-evidence targets. CancerDiscov. (2021) 11(11):2764–79. 10.1158/2159-8290.CD-21-0094 PMC9414287 34373263

[27] Gargallo P Bautista F Juan-Ribelles A Izquierdo E Soriano A de Rojas T Current status of precision medicine in pediatric oncology in Spain: a consensus report by the spanish society of PaediatricHaematology and oncology (SEHOP). ClinTranslOncol. (2022) 24:809–15. 10.1007/s12094-021-02759-7 35152364

[28] Lee DP Skolnik JM Adamson PC. Pediatric phase I trials in oncology: an analysis of study conduct efficiency. J ClinOncol. (2005) 23(33):8431–41. 10.1200/JCO.2005.02.1568 16293874

[29] Kim A Fox E Warren K Blaney SM Berg SL Adamson PC Characteristics and outcome of pediatric patients enrolled in phase I oncology trials. Oncologist. (2008) 13(6):679–89. 10.1634/theoncologist.2008-0046 18586923 PMC6953426

[30] Cohen JW Akshintala S Kane E Gnanapragasam H Widemann BC Steinberg SM A systematic review of pediatric phase I trials in oncology: toxicity and outcomes in the era of targeted therapies. Oncologist. (2020) 25(6):532–40. 10.1634/theoncologist.2019-0615 31943534 PMC7288652

[31] Waligora M Bala MM Koperny M Wasylewski MT Strzebonska K Jaeschke RR Risk and surrogate benefit for pediatricPhase I trials in oncology: a systematic review withmeta-analysis. PLoS Med. (2018) 15(2):e1002505. 10.1371/journal.pmed.1002505 29462168 PMC5819765

[32] Strzebonska K Wasylewski MT Zaborowska L Polak M Slugocka E Stras J Risk and benefit for targeted therapy agents in pediatric phase II trials in oncology: a systematic review with a meta-analysis. Target Oncol. (2021) 16(4):415–24. 10.1007/s11523-021-00822-5 34110559 PMC8266705

[33] Butler E Ludwig K Pacenta HL Klesse LJ Watt TC Laetsch TW. Recent progress in the treatment of cancer in children. CA Cancer J Clin. (2021) 71(4):315–32. 10.3322/caac.21665 33793968

[34] Laetsch TW DuBois SG Glade Bender J Macy ME Moreno L. Opportunities and challenges in drug development for pediatric cancers. Cancer Discov. (2021) 11(3):545–59. 10.1158/2159-8290.CD-20-0779 33277309 PMC7933059

[35] Weinfurt KP Castel LD Li Y Sulmasy DP Balshem AM Benson AB The correlation between patient characteristics and expectations of benefit from phase I clinical trials. Cancer. (2003) 98(1):166–75. 10.1002/cncr.11483 12833469

[36] Moreno L Pearson ADJ Paoletti X Jimenez I Geoerger B Kearns PR Early phase clinical trials of anticancer agents in children and adolescents-an ITCC perspective. Nat Rev ClinOncol. (2017) 14(8):497–507. 10.1038/nrclinonc.2017.59 28508875

[37] Moreno L DuBois SG Glade Bender J Mauguen A Bird N Buenger V Combination early-phase trials of anticancer agents in children and adolescents. J ClinOncol. (2023) 41(18):3408–22. 10.1200/JCO.22.02430 PMC10414747 37015036

