# Supplementary material for: Safety and outcome of children, adolescents and young adults participating in phase I/II clinical oncology trials: a 9-year center experience
Source: Front Pediatr. 2024 Sep 4;12:1423484. doi: 10.3389/fped.2024.1423484 (PMC11421171; doi:10.3389/fped.2024.1423484)
Supplement: Supplementary file 1 [file Table1.pdf]

Supplementary Material

List of clinical trials that have recruited patients in Hospital Niño Jesús

Name	Condition	EudraCT Number
J1S-MC-JP04	Ewing sarcoma	2021-004734-11
I3Y-MC-JPCS	Solid tumors and Neuroblastoma	2019-002931-27
Abraxane	Extracranial solid tumours	2013-000144-26
Alocelyvir	Solid tumors	2019-001154-26
AloCelyvirbrain	Medulloblastoma and DIPG	2020-004838-37
Atezolizumab	Extracranial solid tumours	2014-004697-41
AZA-JMML-001	Myelodisplastic syndrome and juvenile myelomonocytic leukemia	2014-002388-13
BEACON	Neuroblastoma	2012-000072-42
BIOMEDE	Diffuse intrinsic pontine glioma	2014-001929-32
Bosutinib	Chronic myeloid leukemia	2015-002916-34
Carfilzomib (CFZ008)	Acute lymphoblasticleukemia	2014-001633-84
Cobimetinib	Solid tumors	2014-004685-25
ITCC-053 (CRISP)	Anaplastic large cells lymphoma and inflammatory myofibroblastictumour ALK+	2015-005437-53
Dabrafenib (DRB436) and Trametinib (TMT212)	Low or high grade glioma with BRAFV600E mutation-positive	2015-004015-20
Dabrafenib (BRF116013)	Solid tumors with BRAFV600E mutation-positive	2012-001499-12
Daratumumab (JNJ-54767414)	Acute lymphoblastic leukemia or lymphoblastic lymphoma	2017-003377-34
Decitabina (DACOGEN)	Acute myeloid leukemia	2013-000390-70
Durvalumab and Tremelimumab	Solid tumors and hematological malignances	2018-003118-42
Entrectinib	NTRK –ROS fusion positive solidtumors	2019-001155-39
Erbumina (J1O-MC-JZHD)	Neuroblastoma	2019-001042-18

Eribulina (E7389-G000-213)	Solid tumors	2016-003352-67
ESMART	Alltumors	2016-000133-40
GEIS-39	Desmoplatic small round cell tumors and Ewing Sarcoma	2016-002464-14
Glofitamab	Mature B-cell Non Hodgkin Lymphoma	2021-006326-48
Idasanutlin	Acute leukemias or solid tumors	2018-004579-11
ITCC-059 (Inotuzumab)	Acute lymphoblasticleukemia	2016-000227-71
JCAR017	Acute lymphoblasticleukemia	2018-001246-34
LDK 378	All tumors with ALK alteration	2012-002074-31
Lenvatinib OLIE	Osteosarcoma	2013-005534-38
Lenvatinib (MK-7902)	Solid tumors (except osteosarcoma)	2019-004441-33
CH14.18/CHOwith IL2	Neuroblastoma	2009-018077-31
MEMMAT	Medulloblastoma, ependymoma, ATRT	2010-023691-33
Nilotinib	Chronic myeloid leukemia	2013-000200-41
Niraparib/Dostarlimab	Solid tumors	2020-002359-39
Nivolumab and Bempegaldesleukin (CA045-020/19-214-17)	All tumors	2020-000854-85
Nivolumab and Brentuximab vedotin (CA209744)	Classic Hodgkin Lymphoma	2016-002347-41
Nivolumab and lpilimumab (CA209908)	High grade primary CNS malignancies	2016-044441-82
Olaparib	Solid tumors	2018-003355-38
Pazopanib (VEG116731)	Solid tumors	2013-003595-12
Pembrolizumab (MK-3475)	Hodgkin lymphoma	2017-001123-53
Pomalidomida (CC4047)	Primary brain tumors	2016-002903-25
Ponatinib (INCB 84344-102)	Solid tumors	2018-004878-99
Quizartinib (AC220-A-U202)	Acute myeloid leukemia with FLT3-ITD mutations	2016-002919-18
rEECur	Ewing sarcoma	2014-000259-99
Regorafenib (BAY 73-4506)	Solid tumors	2013-003579-36
Selumetinib	Type 1 Neurofibromatosis related Plexyform neurofibroma	2020-005648-52
Surufatinib (2020-012-GLOB2)	Solid tumors	2021-003602-41
Tapistry (BO41932)	Alltumors	2020-001847-16
TVEC	Solid tumors (Non CNS)	2015-003645-25

ITCC-022 (VINILO)	Low grade glyoma	2012-003005-10
ITCC-092 (Vyxeos)	Acute myeloidleukemia	2020-000142-34
YmABS-hU3f8	Neuroblastoma	2017-001829-40

DIPG: diffuse intrinsic pontine glioma; ATRT: atypical teratoid rhabdoid tumor, CNS: Central nervous system
